# Waterfowl occurrence and residence time as indicators of H5 and H7 avian influenza in North American Poultry

**DOI:** 10.1038/s41598-020-59077-1

**Published:** 2020-02-13

**Authors:** John M. Humphreys, Andrew M. Ramey, David C. Douglas, Jennifer M. Mullinax, Catherine Soos, Paul Link, Patrick Walther, Diann J. Prosser

**Affiliations:** 1 Michigan State University, East Lansing, Michigan USA; 2 U.S. Geological Survey, Alaska Science Center, Anchorage, Alaska USA; 3 University of Maryland, College Park, Maryland USA; 4 Environment and Climate Change Canada, Ecotoxicology and Wildlife Health Division, Saskatchewan, Canada; 5 Louisiana Department of Wildlife and Fisheries, Baton Rouge, Louisiana USA; 6 U.S. Fish and Wildlife Service, Texas Chenier Plain Refuge Complex, Anahuac, Texas USA; 7 U.S. Geological Survey, Patuxent Wildlife Research Center, Laurel, Maryland USA

**Keywords:** Ecological epidemiology, Animal behaviour

## Abstract

Avian influenza (AI) affects wild aquatic birds and poses hazards to human health, food security, and wildlife conservation globally. Accordingly, there is a recognized need for new methods and tools to help quantify the dynamic interaction between wild bird hosts and commercial poultry. Using satellite-marked waterfowl, we applied Bayesian joint hierarchical modeling to concurrently model species distributions, residency times, migration timing, and disease occurrence probability under an integrated animal movement and disease distribution modeling framework. Our results indicate that migratory waterfowl are positively related to AI occurrence over North America such that as waterfowl occurrence probability or residence time increase at a given location, so too does the chance of a commercial poultry AI outbreak. Analyses also suggest that AI occurrence probability is greatest during our observed waterfowl northward migration, and less during the southward migration. Methodologically, we found that when modeling disparate facets of disease systems at the wildlife-agriculture interface, it is essential that multiscale spatial patterns be addressed to avoid mistakenly inferring a disease process or disease-environment relationship from a pattern evaluated at the improper spatial scale. The study offers important insights into migratory waterfowl ecology and AI disease dynamics that aid in better preparing for future outbreaks.

## Introduction

Avian influenza is a global concern and poses hazards to human health, food security, and wildlife conservation worldwide^1,2^. Domestic poultry operations are particularly vulnerable to avian influenza viruses maintained in wild bird hosts^3–6^ as the viruses may be spread to poultry^7–10^ via direct contact or by way of environmental contamination. Once introduced into a poultry operation, avian influenza viruses of the H5 and H7 hemagglutinin subtype can rapidly propagate through commercial flocks and mutate to the Highly Pathogenic Avian Influenza (HPAI) pathotype with increased virulence^1,11,12^. HPAI outbreaks can inflict direct stock mortality or necessitate that culling protocols be implemented to minimize the risk of disease spread. In rare instances, control efforts may also reduce the spread of viruses potentially lethal to humans, as was shown for Goose Guangdong (GsGD) lineage HPAI H5N1 and H7N9 in China^13,14^.

Avian influenza viruses circulate among wild aquatic birds globally and taxa such as migratory waterfowl are considered to be natural biologic reservoirs^15^. Waterfowl are infected with varying virus subtypes, including those with the H5 and H7 hemagglutinin protein that have the potential to mutate to HPAI in poultry throughout the Neotropics and Nearctic^16–19^.

During 2014 and 2015, it appears migratory waterfowl contributed to the introduction of GsGD lineage HPAI H5 viruses into North America^20–22^ and subsequent spread in Canada and the U.S.^23–26^. This outbreak was the largest in U.S. history, affected wild and domestic birds in 21 U.S. States, resulted in the loss of approximately 50 million poultry, and caused estimated losses of more than 3 billion US dollars^27,28^.

To assess the potential role of migratory waterfowl in transporting HPAI viruses throughout central and eastern Asia, several studies have examined the spatial and temporal overlap of waterfowl movement trajectories or utilization distributions with disease occurrence locations, poultry facilities, and key waterfowl habitats^3,29–33^, however, there are a lack of comparable waterfowl movement studies investigating associations with avian influenza outbreaks in the Western Hemisphere. Indeed, there is a recognized need for new methods and tools to help quantify the dynamic interaction between wild bird hosts and commercial poultry in North America^34–36^. To help address this knowledge gap, we developed a combined animal movement and disease distribution model to evaluate the spatiotemporal relationship of migratory waterfowl to H5 and H7 subtype avian influenza detections in commercial poultry (AIPs) within North America.

For this study, we define AIPs as being any documented outbreak or infection caused by H5 or H7 subtype avian influenza in commercial poultry. Our central objective was to leverage multi-year animal movement telemetry and poultry disease data to characterize intra-annual patterns within a complex disease system. To accomplish this task, we used satellite-marked blue-winged teal (*Anas discors*) as a representative sample of the larger North American dabbling duck population and applied Bayesian joint hierarchical modeling to concurrently model species distributions, residency times, migration timing, and AIP occurrence probability. Blue-winged teal were chosen as a representative waterfowl species due to their widespread distribution and suspected role in redistributing avian influenza viruses throughout breeding grounds in the U.S. Northern Great Plains and Canada and overwintering habitats in Mexico^17^, the Caribbean^37^, and Central and northern South America^16,18,38^. Because migratory behavior and habitat preferences can differ between duck species^39^, the selection of blue-winged teal as an archetypal dabbling duck species necessitates that several important caveats be considered. As examples, blue-winged teal have a tendency to start the spring migration later, undertake the fall migration earlier, and fly greater distances than other dabbling ducks^40,41^. These caveats are examined in the context of results’ interpretation in the closing discussion.

Essential to our proposed method was taking steps to ensure that spatial correlation between satellite-marked birds and AIP locations did not bias model results and that the inclusion of locally-abundant (clustered) AIP records did not inflate model estimates or predictions. AIP records that are aggregated in time or space may signify non-typical increases in disease incidence that offer special insight into the system’s ecology. Recognizing that disease clusters can be difficult to interpret and statistically problematic, we took steps to avoid mistakenly inferring a disease process or disease-environment relationship from a pattern evaluated at the improper spatial scale^42,43^. In the case of poultry AIPs, localized clusters may be borne of epidemiological mechanisms operating at a much finer spatial scale than those patterns arising from long-range viral dispersion by migratory birds^44^. For instance, failures in biosecurity may result in the unintentional transmission of viral pathogens among farms via contaminated farm equipment long after initial introduction by a wild bird^26,28,45^. To better differentiate between possible farm-to-farm transmission and novel wild bird introduction, our model was designed to accommodate multi-scaled spatial processes.

## Materials and Methods

### Animal protocols statement

The authors confirm that all methods were carried out in accordance with relevant guidelines and regulations and that all experimental protocols were approved by the appropriate institutions and licensing committees. Protocols for capturing, handling, and instrumenting blue-winged teal included in this study were reviewed by institutional animal care and use committees (USGS Alaska Science Center Animal Care and Use Committee Animal Use permit no. 2014–5 and the University Committee on Animal Care and Supply University of Saskatchewan Animal Use protocol no. 20070039) and carried out under federal authority (US Department of the Interior Federal Bird Banding permit nos. 09072 and 23792, Federal Fish and Wildlife permit no. MB779238-2, Environment Canada Migratory Bird Banding permit no. 10458R).

### Study domain and bird telemetry

Our study area encompassed North America between 14.5° and 60.0° North Latitude and −137.5° and −66.2° West Longitude. This area includes the conterminous United States, Mexico, Cuba, the Bahamas, and major portions of Canada. Forty-two adult, male blue-winged teal were captured and marked. Twelve birds were captured on their summer range in the Saskatchewan and Alberta Canadian Provinces during August 11–15, 2013, with the remaining thirty birds captured at wintering areas along the Texas and Louisiana Gulf Coast in the United States during March 17–22, 2015. All birds were marked with 9.5 gram, solar-powered Platform Transmitter Terminals (PTTs) manufactured by Microwave Telemetry, Inc., Columbia, Maryland (USA) and then released in close proximity to their respective capture locations. The PTTs were attached using a Teflon harness secured over the sternum to have a dorsally extended antenna as outlined by Takekawa *et al*.^46^. The PTTs were programmed to have a regular duty cycle with a 10 hour on period every 48 hours. We used the Argos Data Collection and Location System (CLS America, Incorporated, Largo, Maryland, USA) to receive transmissions, which provided Doppler-based location estimates, a movement activity indicator, and a class index reporting the quality of each location estimate. To prepare telemetry for statistical modeling, we first applied a filtering algorithm^47^ and then manually inspected movements and activity sensor records to exclude dates following likely bird mortality or PTT detachment. Locations exhibiting an unchanged activity indicator over two or more duty cycles were removed from the telemetry dataset. Table 1 details the bird-specific number of locations retained for analysis following data cleaning. Animations depicting the wild bird’s used in this study and downloadable telemetry data are available from the U.S. Geological Survey, Alaska Science Center, Wildlife Tracking Data Collection (10.5066/P9Z9BA9F)^48^.

**Table 1:** Telemetry summary.

Tag	Deployment	Last	Mortality	Duration	Median	Sample	Latitude	Longitude	Region
131009	8/13/2013	10/26/2013	Hunting	74	29	558	52.04	−107.10	Saskatchewan
131010	8/13/2013	3/26/2014	—	225	40	1142	52.04	−107.10	Saskatchewan
131011	8/14/2013	4/27/2015	—	620	48	2730	50.48	−112.12	Alberta
131012	8/14/2013	11/5/2013	—	83	41	464	50.48	−112.12	Alberta
131013	8/14/2013	10/24/2013	Hunting	71	37	453	52.04	−107.10	Saskatchewan
131014	8/11/2013	10/24/2013	—	73	31	523	52.04	−107.10	Saskatchewan
131015	8/12/2013	4/8/2014	—	239	43	1149	52.04	−107.10	Saskatchewan
131016	8/12/2013	1/25/2014	—	165	42	803	52.04	−107.10	Saskatchewan
131017	8/12/2013	10/21/2013	—	70	32	516	52.04	−107.10	Saskatchewan
131018	8/12/2013	10/17/2013	—	66	32	468	52.04	−107.10	Saskatchewan
131019	8/15/2013	11/29/2013	—	106	42	593	50.48	−112.12	Alberta
131020	8/15/2013	10/17/2013	—	63	39	408	50.48	−112.12	Alberta
131009	3/17/2015	10/25/2015	—	222	46	1087	30.01	−94.14	Texas
131013	3/19/2015	9/26/2015	—	191	47	898	29.61	−94.53	Texas
135841	3/17/2015	12/9/2015	Hunting	267	49	1165	30.01	−94.14	Texas
135842	3/17/2015	11/12/2015	—	240	57	816	30.01	−94.14	Texas
135843	3/17/2015	10/31/2015	—	228	47	1055	30.01	−94.14	Texas
135844	3/17/2015	5/28/2015	—	72	49	335	30.01	−94.14	Texas
135845	3/18/2015	4/28/2015	—	41	52	181	29.67	−94.44	Texas
135846	3/18/2015	10/2/2015	Hunting	198	43	1061	29.67	−94.44	Texas
135847	3/18/2015	8/17/2015	—	152	56	565	29.67	−94.44	Texas
135848	3/18/2015	10/27/2015	—	223	46	1051	29.67	−94.44	Texas
135849	3/18/2015	9/27/2016	—	559	53	2129	29.67	−94.44	Texas
135850	3/19/2015	3/31/2015	—	12	53	57	29.61	−94.53	Texas
135851	3/19/2015	5/11/2015	—	53	54	205	29.61	−94.53	Texas
135852	3/19/2015	9/30/2016	—	561	56	1918	29.61	−94.53	Texas
135853	3/19/2015	5/5/2015	—	47	53	211	29.61	−94.53	Texas
135854	3/19/2015	10/18/2015	—	213	48	938	29.61	−94.53	Texas
135855	3/22/2015	10/21/2015	—	213	48	955	30.55	−91.85	Louisiana
135856	3/22/2015	7/12/2015	—	112	48	532	30.55	−91.85	Louisiana
135857	3/22/2015	7/22/2015	—	122	51	519	30.55	−91.85	Louisiana
135858	3/22/2015	5/8/2015	—	47	47	226	30.55	−91.85	Louisiana
135859	3/22/2015	7/17/2015	—	117	46	569	30.55	−91.85	Louisiana
135860	3/22/2015	12/23/2016	—	642	50	2647	30.55	−91.85	Louisiana
135861	3/22/2015	4/13/2016	—	388	50	1338	30.55	−91.85	Louisiana
135862	3/22/2015	8/14/2015	—	145	55	528	30.55	−91.85	Louisiana
135863	3/22/2015	4/17/2015	—	26	50	125	30.55	−91.85	Louisiana
135864	3/21/2015	10/28/2015	Hunting	221	48	1019	30.46	−91.57	Louisiana
135865	3/21/2015	4/7/2015	—	17	51	89	30.46	−91.57	Louisiana
135866	3/21/2015	5/31/2015	—	71	53	290	30.46	−91.57	Louisiana
135867	3/21/2015	11/23/2015	—	247	47	1141	30.46	−91.57	Louisiana
135868	3/21/2015	4/7/2015	—	17	49	84	30.46	−91.57	Louisiana

The PTT transmitter identifier (Tag), date of deployment (Deployment), last transmission date (Last), bird mortality type if known (Mortality), duration of record (Duration, days), median sampling interval between locations (Median, minutes), total locations (Sample), and the coordinates (Latitude, Longitude) and geographic region (Region) of deployment for records retained for analysis.

### Environmental data

To characterize habitat and environmental conditions over the study area, we utilized 250 meter resolution gridded soils data^49^, the Global Topographic 30 Arc-Second Digital Elevation Model available from the U.S. Geological Survey (USGS) Earth Resources Observation and Science Center (https://www.usgs.gov/centers/eros), the 300 meter 2009 GlobCover Land Cover Maps provided by the European Space Agency (http://due.esrin.esa.int/page_globcover.php), and 30 meter resolution topographic roughness and topographic wetness indices provided by the ENVIREM dataset^50^. Because our goal with incorporating environmental variables was to characterize waterfowl habitat broadly and not to infer species-specific preferences or tolerances, we decomposed gridded soils data (17 different soil attributes) and elevation derived variables (topographical roughness, topographical wetness, elevation, and slope) using separate Principal Components Analyses and selected the resulting first component from each to represent soil and topographical conditions respectively. The correlation of the retained first component to other attributes included during decomposition are provided for soils in Table 2 and topography in Table 3. In addition to simplifying our analysis, the decomposition of variables also aided in avoiding multicollinearity issues during the model fitting process^51^. Recognizing the critical importance of wetlands and surface waters to waterfowl, we also calculated the Euclidean distance (km) of each telemetry location to the nearest surface water class in the GlobCover land cover dataset using the Program R^52^ and the **spatstat** package^53^. As detailed in subsection 2.6, we modeled distance to water as a non-linear covariate to avoid treating water presence as a bird occurrence prerequisite.

**Table 2:** Soils analysis.

Attribute	r (%)	Description
AWCh1	−0.21 (0.01)	Available water capacity (Horizon 1)
AWCh2	−0.20 (0.01)	Available water capacity (Horizon 2)
AWCh3	−0.20 (0.01)	Available water capacity (Horizon 3)
AWCtS	−0.46 (0.03)	Saturated water content
BLDFIE	0.48 (0.03)	Bulk density (kg/m^3^)
CECSOL	−0.23 (0.01)	Cation exchange capacity (cmolc/kg)
CLYPPT	0.18 (0.00)	Clay content mass fraction
CRFVOL	−0.68 (0.15)	Coarse fragments volumetric
OCDENS	0.20 (0.59)	Organic carbon density (kg/m^3^)
OCSTHA	−0.21 (0.02)	Organic carbon stock (tons/ha)
ORCDRC	−0.32 (0.06)	Organic carbon content (g/kg)
PHIHOX	0.28 (0.03)	Soil pH × 10 (H_2_O)
PHIKCL	0.23 (0.01)	Soil pH × 10
SLTPPT	0.38 (0.02)	Silt content mass fraction
SNDPPT	−0.44 (0.01)	Sand content mass fraction
TEXMHT	−0.08 (0.01)	Texture class (USDA system)
WWP1	−0.15 (0.00)	Available water capacity (wilting point)

Correlation of 17 soil attributes to decomposed component retained as soil covariate. Soil attribute name (Attribute), Pearson correlation (r) with parenthetical proportion of variance explained (%), and attribute description. All correlations were significant at the $\alpha =0.001$ threshold.

**Table 3:** Topographical analysis.

Attribute	r (%)
Slope	−0.51 (0.00)
Elevation	−0.59 (0.01)
Compound Topographic Index	0.93 (0.81)
Terrain Roughness Index	−0.52 (0.18)

Correlation of 4 topographical attributes to decomposed component retained as a topographical covariate. Attribute name (Attribute), Pearson correlation (r) with parenthetical proportion of variance explained (%), and attribute description. All correlations were significant at the $\alpha =0.001$ threshold.

### Poultry and human density data

**Figure 1:**
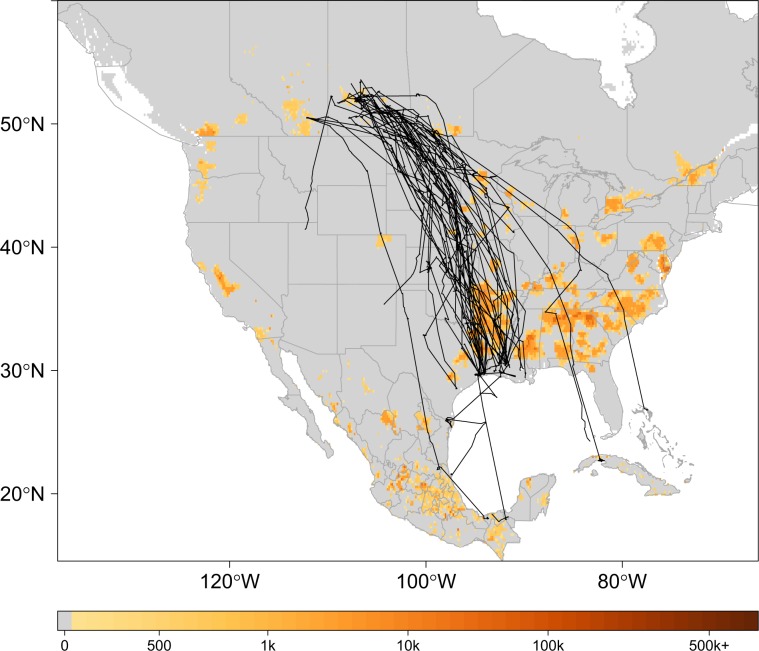
Track Map. Telemetry tracks for 42 blue-winged teal over grid depicting poultry abundance^54^. Black lines show individual bird tracks, legend describes estimated number of poultry (chickens and ducks) per km^2^. To better display poultry abundance in this figure, zero was defined as less than 100 poultry/km^2^.

For estimates of poultry abundance, we used the chicken and duck raster layers from the Food and Agriculture Organization of the United Nations (FAO) Gridded Livestock database^54^. The rasters were provided at a 0.083333 decimal degree resolution and report the number of poultry head per square kilometer (head/km^2^). Human population density estimates were obtained as a continuous raster (30 meter) from NASA’s Socioeconomic Data Center that gave the number of persons per square kilometer (persons/km^2^)^55^. The distribution of poultry abundance relative to our study domain is shown in Fig. 1 with telemetry tracks overlaid for marked birds.

### Avian influenza event in poultry data

**Figure 2:**
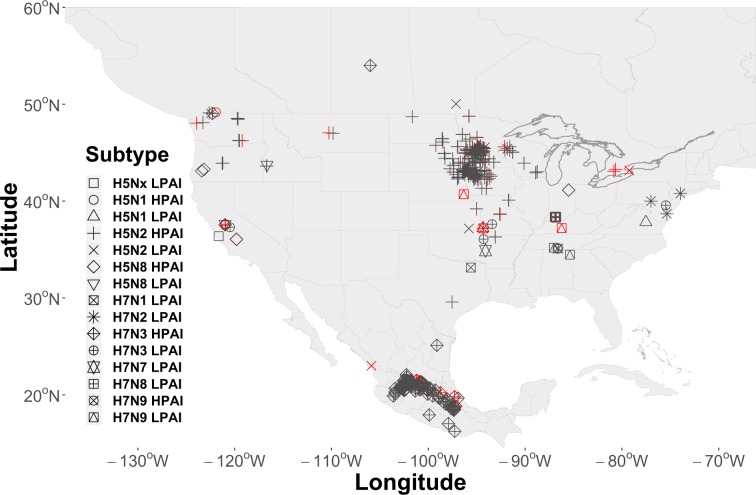
Locations of Avian Influenza Events (AIP) by virus subtype. AIP cover the period 2004–2019 and included 440 events used for model training and 49 (20%) randomly selected for model validation. Locations shown in the color red signify those retained for validation.

AIP data were obtained from the web-based Global Animal Disease Information System (EMPRES-i) maintained by the FAO (http://empres-i.fao.org/eipws3g/, accessed 12/01/19). EMPRES-i is intended to support veterinary services and related organizations by providing access and analysis of global animal disease information. We initially downloaded all AIP data for the period between January 01, 2004 and December 01, 2019 and then filtered records to include only those with an FAO “Confirmed” status, geographically located within our study domain, affecting commercial poultry, and associated with virus subtypes having a H5 or H7 hemagglutinin surface protein. The H5 and H7 subtypes were chosen due to our interest in the HPAI pathotype, however, both HPAI (n = 440) and LPAI (n = 49) events were included in the resulting 489 record dataset. A secondary consideration in limiting the study to the H5 and H7 subtypes was that these subtypes are required to be reported to the World Organization for Animal Health (OIE) giving us some confidence in our sample. This is not the case with other virus subtypes, which are reported voluntarily and may therefore introduce sample bias. As a final step in preparing AIP data, we randomly selected 97 AIPs (approximately 20%) as a hold-out dataset to aid in later model validation. Locations of all events are shown by virus subtype in Fig. 2.

### Statistical analysis

We constructed a two-level, spatially explicit model with shared components to evaluate the spatial and temporal relationship of waterfowl to AIPs across North America. The model’s first level estimated waterfowl residence time across the study area, whereas, the model’s second level provided the probability of an AIP as conditioned on that waterfowl residence time. More generally, our model can be represented as:

1 $${y}_{1}(i)={\alpha }_{1}+\beta \cdot {X}_{1}(i)+{Z}_{1}(i)+{e}_{1}(i)$$

2 $${y}_{2}(i)|{y}_{1}(i)={\alpha }_{2}+\beta \cdot {X}_{2}(i)+{Z}_{2}(i)+{Z}_{shared}(i)+{e}_{2}(i)$$

where the first level [Eq. 1] estimates waterfowl residence time (*y*_1_) at locations *i* $(i=1,2,3,\ldots ,n)$ and the second level [Eq. 2] gives AIP probability (*y*_2_) based on the past event (H5 or H7 AI detection) occurrence or absence (1, 0) at location *i* and conditioned on waterfowl residence time (${y}_{1}$). This formulation offers independent intercepts for each model level (${\alpha }_{j}$), covariate matrices ($\beta \cdot {X}_{j}$) for level-specific linear and fixed effects, and uncorrelated error terms (${e}_{j}$). Although independent spatial fields (${Z}_{1}$ and ${Z}_{2}$) help account for spatial dependencies within each model level, we added a third spatial field (${Z}_{shared}$) to explicitly serve as a shared scaling parameter between model levels^56,57^. The shared field is a re-scaled copy of ${Z}_{1}$ that controls for spatial autocorrelation and interaction occurring between waterfowl telemetry and AIP locations. As an initial step in specifying the spatial fields, we created a triangulated mesh over our study area using procedures described by Lindgren and Rue^58^. In addition to facilitating parameterization of the continuous spatial fields needed to account for spatial dependencies, the mesh provided a computationally efficient alternative to characterizing our continent-sized study domain using raster grids or areal polygons. The mesh included 9,555 nodes and had an outer extension large enough to avoid edge effects around study area boundaries. Due to our large study area, the mesh was projected using three-dimensional Cartesian coordinates scaled to one Earth radius (6,371 km). As a component of sensitivity analyses, meshes constructed with 4,897 nodes and 12,651 nodes were also evaluated and found to exhibit a mean spatial effect within 10% of that estimated using 9,555 nodes; therefore, we selected the middle-ground to balance precision and processing time.

To accommodate the joint modeling approach, our dependent variable was structured as a bivariate matrix with the first column corresponding to residence times calculated from telemetry data and the second column being a vector of 1’s and 0’s designating a past AIP occurrence (1) or absence (0) at each location. Because bird and AIP locations did not always geographically coincide, our model incorporated spatial misalignments; however, this was not problematic as the model is capable of providing estimates across continuous space and for all locations within the study domain^56^. To acquire initial residence time estimates for model fitting, we disaggregated individual bird telemetry tracks (Fig. 1) into 1 minute temporal intervals and then summed them based on mesh node proximity (i.e., “natural neighborhoods”). To achieve this, we performed a Voronoi tessellation around mesh nodes to identify each node’s respective area of influence^56^. Area of influence refers to the bounded area surrounding each node in which all enclosed locations share that node as their closest point^59^. The process used to disaggregate tracks into equal time intervals was adapted from Sumner^60^, but, modified to allow for summation of time intervals based on our tessellated neighborhoods as opposed to a regular grid. Each column of the bivariate matrix exhibited a different distribution, therefore, we specified model likelihoods as,

$${y}_{1}(i)\sim {\rm{Gamma}}({a}_{i},{b}_{i})\,\text{and}\,\,{y}_{2}(i)\sim {\rm{Binomial}}({\pi }_{i})$$

where, residence time was always a positive value with shape and scale parameters (${a}_{i},{b}_{i}$) such that ${a}_{i}/{b}_{i}={\mu }_{i}=E({v}_{i})\cdot Exposure$. Defining *Exposure* as the area of each node’s tessellated neighborhood, the residence-time linear predictor ${v}_{i}$ was of the form,

3 $$\begin{array}{rcl}\log ({v}_{i}) & = & {\alpha }_{1}+{\beta }_{1}\,{{\rm{Soil}}}_{i}+{\beta }_{2}\,{{\rm{Topography}}}_{i}\\  &  & +\,{\beta }_{3}\,{{\rm{WetDistance}}}_{i}+{Z}_{1}(i).\end{array}$$

here, a log link function was used with ${\alpha }_{1}$ as the intercept, ${\beta }_{1}$ the soil variable coefficient, ${\beta }_{2}$ the topography coefficient, ${\beta }_{3}$ the coefficient for linear distance to nearest wetland, and ${Z}_{1}$ as the spatial field to control for spatial dependencies and other unobserved latencies (errors) that may have resulted from the aggregating process undertaken to estimate residence time.

The corresponding linear predictor for the binomially distributed AIP occurrence vector relied on the logit function and was specified as,

4 $$\begin{array}{rcl}{\rm{logit}}({\pi }_{i}) & = & {\alpha }_{2}+{\beta }_{1}\,{{\rm{Poultry}}}_{i}+{\beta }_{2}\,{{\rm{Population}}}_{i}\\  &  & +\,{\beta }_{3}\cdot {f}_{1}\,{{\rm{Cluster}}}_{i}+{\beta }_{4}\cdot {f}_{2}\,{{\rm{Displacement}}}_{i,t}\\  &  & +\,{Z}_{2}(i)+{Z}_{shared}(i),\end{array}$$

where ${\alpha }_{2}$ is an intercept, ${\beta }_{1}$ is the coefficient for poultry density and ${\beta }_{2}$ is the human population density coefficient. As described for Eq. 2, ${Z}_{2}$ provides a spatial field to account for domain-wide spatial dependencies and latencies among AIP locations, whereas, ${Z}_{shared}$ controls for the spatial relationship between AIP and waterfowl locations. To supplement poultry and human population density variables, we developed two additional model covariates; one to account for fine-scale spatial structure among AIP locations $({f}_{1}\,{\rm{Cluster}})$ and the second to examine the temporal relationship between seasonal waterfowl migration and the timing of AIPs $({f}_{2}\,{\rm{Displacement}})$.

As shown by Fig. 2, the geographic distribution of AIPs across North America exhibits broad regional coverage (e.g., the Midwestern U.S.) as well as more localized or concentrated “clustering“ at some locations (e.g., Jalisco, Mexico or Minnesota, USA). We speculate that the epidemiological processes that gave rise to the fine-scaled, cluster patterns were likely different than those that instigated the broad-scale, region-wide coverage. More specifically, it is possible that farm-to-farm transmission or “lateral spread” produced the observed clusters, whereas, the broader coverage may be better explained by waterfowl introduction (see, Discussion for further explanation). We chose to address these different multi-scaled spatial processes using two separate spatial covariates. The spatial field (${Z}_{2}$) was used as a domain-wide spatial covariate, while fine-scale spatial structure associated with AIP clusters was modeled using a random walk process across AIP nearest neighbor distances (km). As used here, a random walk $({f}_{1}\,{\rm{Cluster}})$ is similar to a regression spline in that it permits distances between AIPs to follow a smooth, curvilinear distribution without assuming a mean response. The benefit of using the fine-scale spatial effect is that it quantifies the spatial variability due to proximity between AIP locations, thereby, reducing the model’s capacity to attribute that variation to waterfowl or other covariates.

**Figure 3:**
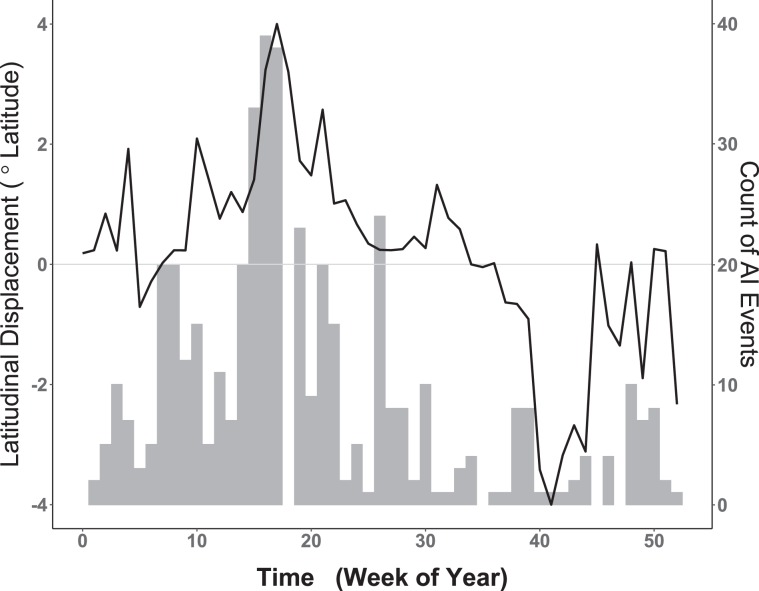
Weekly comparison of historic AI Events (AIPs) with waterfowl latitudinal displacement. Left vertical axis corresponds to smooth black line and represents weekly latitudinal displacement of waterfowl measured in degrees latitude. Horizontal axis provides the week of year. Dotted line intersecting zero signifies no net waterfowl displacement, with portions above zero indicating relative northward movement and portions below showing net southward change. Vertical axis at right corresponds to gray histogram for the weekly counts of all AIP summed over the period 2004–2019. Note apparent temporal correlation between waterfowl spring migration (Weeks 5–18) and increasing AIP as well as that between fall migration (weeks 35–42) and decreased AIP counts.

As with clustering, a random walk was also implemented to quantify the temporal relationship between seasonal waterfowl migration and the timing of AIPs. As shown in Fig. 3, summing raw AIP counts (all years, 2004–2019) by week reveals an apparent correlative relationship of AIP timing to the mean weekly latitudinal displacement exhibited by waterfowl. To capture the timing of waterfowl movement as a model covariate, we calculated latitudinal displacement by finding the weekly mean latitude occupied by marked birds (all years, 2013–2016) and then performing a one-week lag subtraction between weeks. The function ${f}_{2}\,{\rm{Displacement}}$ introduces the resulting time-ordered vector as a non-parametric, dynamic effect that captures waterfowl latitudinal change at each weekly time step *t* $(t=1,2,3,\ldots ,52)$.

We used a Penalized Complexity (PC) framework^61,62^ for the three spatial fields $({Z}_{j})$ with priors scaled to one Earth radius to reflect the size and projection of our 3D triangulated mesh. Because all spatial fields occurred over a common mesh and spatial domain, PC priors were set with both the spatial range and standard deviation quantile and probability: ${\rho }_{0}=1$ and ${p}_{\rho }=0.5$. The PC priors for the spatial effect were intended as non-informative and can be interpreted as specifying a 0.5 probability that the spatial range is within 6,371 km (i.e., one Earth radius). Sensitivity analyses for probability values between 0.2 and 0.8 in 0.1 increments assuming a ${\rho }_{0}=1$ revealed minimal change to model estimates. Flat PC priors were likewise applied for the random walk effects modeled as ${f}_{1}\,{\rm{Cluster}}$ and ${f}_{2}\,{\rm{Displacement}}$, which were specified as $\mu =1$ and $\alpha =0.0001$. We then combined priors and likelihoods using Integrated Laplace Approximation^63^.

In addition to performing joint estimation of waterfowl residence time and AIP occurrence probability as described above, we also explored an alternative model formulation to jointly estimate waterfowl occurrence probability with AIP occurrence probability. That is, rather than using residence time to inform AIP probability, the alternate formulation used mere waterfowl occurrence to estimate AIP probability as a way of comparing the relative importance of bird presence versus the amount of time spent at a location. Under the alternative model, we assumed that all locations with a residence time equal to, or exceeding 24 hours constituted an occurrence (1) with unobserved locations (0) used to characterize background environmental conditions. To correspond with the derived presence-background vector, we substituted a binomial likelihood for the gamma in Eq. 3, and exchanged a logit link function for the log. The alternative binomial-binomial configuration included all of the same spatial and environmental covariates as described for the Gamma-binomial.

### Model evaluation

To evaluate model accuracy, we performed model-based estimation for each AIP location and event time in the independent testing dataset that was randomly selected and excluded from model development. We calculated the percent correctly classified (PCC), sensitivity (proportion of correctly predicted presences), specificity (proportion of correctly predicted absences), and the area under the receiver operating characteristic curve (AUC) using the Presence Absence R package^64^, and then estimated the True Skill Statistic (TSS) by subtracting a value of one from the sum of the sensitivity and specificity estimates^65^. We also used tools available in the Presence Absence package to determine the probability thresholds that best maximize sensitivity, specificity, and the TSS. Probability thresholds were determined by comparing the input data to model estimates to ascertain the probability value (threshold) that best captured known disease occurrences.

In addition to validating with an independent out-of-sample testing data set, we applied a comparative approach to assess the overall performance of our joint waterfowl residence time and AIP occurrence probability model. In total, seven different models were constructed and then compared to the full joint model detailed in subsection 2.6, one of which (Model6) was the alternative formulation described in the closing paragraph of subsection 2.6. Recognizing that application of parsimony metrics to spatial models can be problematic^66,67^, we chose three different parsimony measures; the deviance information criterion (DIC), the Watanabe-Akaike information criterion (WAIC), and the log-conditional predictive ordinate (lCPO). Generally, the DIC and WAIC are comparable, however, the DIC sometimes under-penalizes random effects^67^; thus, we opted to use both the DIC and WAIC as well as leave-one-out cross-validation (lCPO). Descriptions for all comparative models are provided with accompanying DIC, WAIC, and lCPO in the results section.

## Results

**Figure 4:**
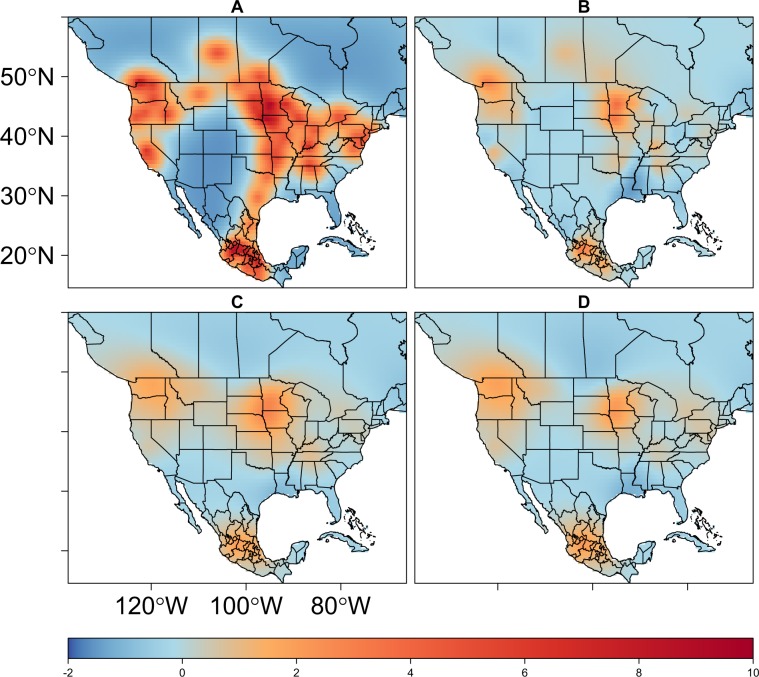
Random field density comparison. Spatial field resulting from the (**A**) base model (Model1), (**B**) base model with clustering effect and other covariates, (**C**) joint-model for residence time (Model5), and (**D**) joint-model for occurrence (Model6). Red areas highlight where models under predict AIP, cooler blue regions indicate where models over predict. Maps are plotted on the same scale using the same palette and can be interpreted as “mapped residuals”. Note that random field densities for models incorporating telemetry (**C**,**D**) are reduced relative to models without telemetry (**A**,**B**), but, even the telemetry models underestimate AIPs over several areas in the upper Midwestern and Northwestern U.S., as well as in central Mexico. Residual error indicates that additional factors or variables are needed to fully explain the AIPs at these locations.

Table 4 lists all models used to assess performance relative to the full model described in subsection 2.6. Note that in Table 4, the DIC, WAIC, and lCPO indicate improved parsimony as additional covariates were added, and that joint models combining waterfowl telemetry (Model5 and Model6) with AIP occurrences exhibited the best overall performance. As a visual comparison, Fig. 4(A) maps the smoothed random field densities produced by the base spatial model with no covariates (Model1) in comparison to a spatial model with all covariates, but, excluding waterfowl telemetry (Model4, Fig. 4(B)), and the joint models incorporating waterfowl residence time (Model5, Fig. 4(C)) and occurrence probability (Model6, Fig. 4(D)). Each of the sub-figures in Fig. 4 display a zero mean domain density and can be interpreted in a manner similar to mapped spatial residuals. The map’s warm colors (oranges and reds) indicate regions where actual values are greater than those estimated by the model and cool colors (blues) highlight regions where actual values fall below those estimated by model. Note that random field densities for both joint models are reduced relative to those resulting from Model1 and Model4, but, the joint models still underestimate AIPs over areas in the upper Midwestern and Northwestern U.S., as well as central Mexico. Residual error indicates that additional factors or variables are needed to fully explain the AIPs at these locations.

**Table 4:** AIP model comparison.

Model	DIC	WAIC	lCPO	Description
Model1	2134.42	1963.92	0.178	Space Only
Model2	1247.12	1215.90	0.062	Space + Clustering
Model3	1114.29	1084.77	0.054	Space + Time
Model4	1039.38	1019.96	0.051	Space + Time + Covariates
Model5	842.08	824.30	0.043	Joint Model (Duration)
Model6	836.47	821.35	0.042	Joint Model (Occurrence)
Model7	3710.55	3715.98	0.184	Non-Spatial + Covariates

Deviance information criterion (DIC), Watanabe-Akaike information criterion (WAIC), and log-conditional predictive ordinate (lCPO). Lower values indicate improved parsimony. Joint models displayed in this table, metrics reflect the AIP occurrence probability level of the model only (Eq. 2).

**Figure 5:**
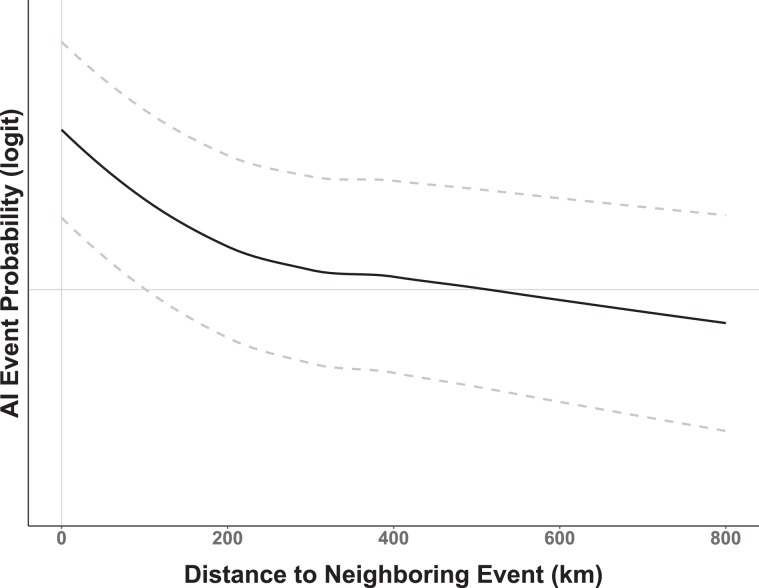
Model estimated clustering covariate. Non-linear, smooth line describes AIP probability as a function of proximity to other, adjacently located AIP occurrences. Vertical axis provides probability (logit scale) and the horizontal axis gives distance in kilometers. Dashed gray lines display 95% Credible Interval. Note that the credible interval includes zero at distances greater than approximately 170 km.

**Figure 6:**
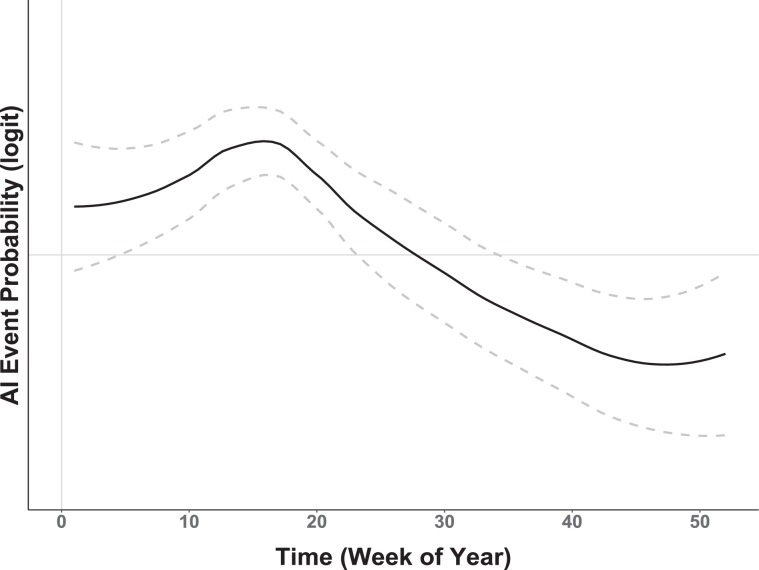
Model estimated temporal effect. Non-linear response of AIP probability as a function of time-structured bird latitudinal displacement. Vertical axis provides probability (logit scale) and the horizontal axis gives time in weeks. Dashed gray lines display 95% Credible Interval. Note that the credible interval includes zero between Weeks 20–35, a time period corresponding with the waterfowl breeding season.

Tables 5 and 6 summarize model covariates for the waterfowl residence time and occurrence probability models jointly fit with AIP occurrence probability. Observe that the soils covariate is important as judged by its credible intervals for both Model5 and Model6, whereas, the topography covariate is not. The soils and topography covariates likely correlate to waterfowl habitat preferences, but, this variation is better captured by soils than by topography. Soils and topography have been successfully used to model wetland occurrence probability in other studies^68^. The distance to water covariate is negative for both models suggesting that as distance from water increases, both waterfowl occurrence probability and waterfowl residence time decrease. Coefficients for poultry abundance are similar across models signifying an approximate 52.9% [(1/(1 + exp(0.12))) × 100%] increase in AIP occurrence probability for every additional 100,000 poultry head/km^2^ above the domain average while holding all other covariates constant. The ${f}_{1}\,{\rm{Cluster}}$ effect is plotted in Fig. 5 and indicates that the probability of an AIP occurrence increases non-linearly as proximity to a known AIP decreases below approximately 300 km. More simply, the closer a location is to a detection of H5 or H7 subtype influenza virus, the more likely it is to be subject to an AIP itself. The lower credible interval in Fig. 5 is inclusive of zero at distances greater than about 170 km, meaning that there is a low posterior probability of equal occurrence odds at longer distances. Figure 6 displays the functional relationship of AIP occurrence to time-structured waterfowl latitudinal displacement. The effect is important over most of the year and suggests that the greatest probability of an AIP occurs at approximately the 18th week of the year (late April - early May), a period largely coinciding with the peak of northward mean waterfowl latitudinal displacement (spring migration, see Fig. 3).

**Table 5:** Joint model (Model5, residence time) effects and hyperparameters.

	Mean	SD	Q025	Q975
Intercept1 (*α*_1_, Eq. 3)	1.66	0.24	1.18	2.13
Intercept2 (*α*_2_, Eq. 4)	−8.06	1.2	−10.42	−5.71
Soils	0.19	0.07	0.06	0.32
Topography	−0.10	0.18	−0.47	0.27
Distance to wetland	−0.02	0.01	−0.04	−0.01
Poultry abundance	0.12	0.03	0.06	0.18
Human population	0.05	0.01	0.02	0.07
Gamma precision	2.17	0.27	1.70	2.73
Range *Z*_1_	0.09	0.03	0.05	0.15
SD *Z*_1_	0.89	0.15	0.64	1.21
Range *Z*_2_	0.25	0.10	0.12	0.50
SD *Z*_2_	1.39	0.39	0.81	2.32
${f}_{1}\,{\rm{Cluster}}$	0.10	0.04	0.05	0.19
${f}_{2}\,{\rm{Displacement}}$	0.76	0.27	0.36	1.41
*Z*_*shared*_	0.56	0.25	0.05	1.04

Mean, standard deviation (SD) and 95% Credible Interval. Coefficients are on the logit scale (logarithm of odds ratio).

**Table 6:** Joint model (Model6, occurrence probability) effects and hyperparameters.

	Mean	SD	Q025	Q975
Intercept1 (*α*_1_, Eq. 3)	−6.15	0.64	−7.40	−4.90
Intercept2 (*α*_2_, Eq. 4)	−8.02	1.20	−10.37	−5.67
Soils	0.36	0.14	0.09	0.65
Topography	0.23	0.30	0.36	0.81
Distance to wetland	−0.03	0.01	−0.05	−0.01
Poultry abundance	0.12	0.03	0.06	0.19
Human population	0.05	0.01	0.02	0.07
Range *Z*_1_	0.15	0.04	0.09	0.25
SD *Z*_1_	3.98	0.66	2.86	5.45
Range *Z*_2_	0.25	0.10	0.12	0.51
SD *Z*_2_	1.35	0.38	0.79	2.26
${f}_{1}\,{\rm{Cluster}}$	0.10	0.04	0.04	0.19
${f}_{2}\,{\rm{Displacement}}$	0.77	0.27	0.36	1.42
*Z*_*shared*_	0.13	0.08	0.04	0.30

Mean, standard deviation (SD) and 95% Credible Interval. Coefficients are on the logit scale (logarithm of odds ratio).

**Figure 7:**
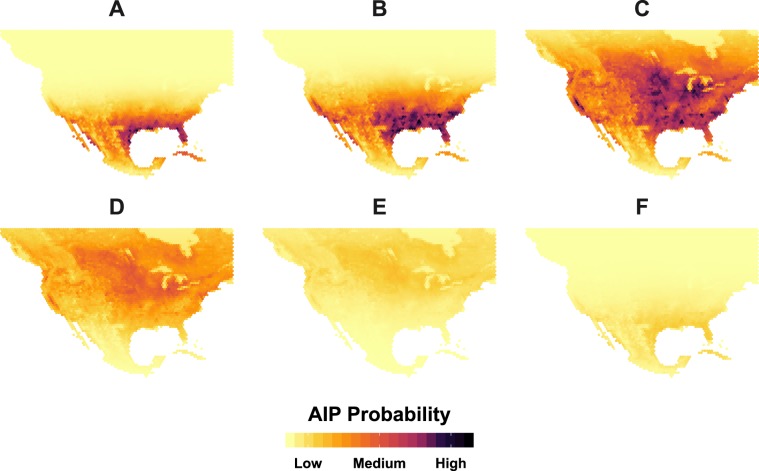
Mapped AIP probabilities predicted by joint-model (Model5). Weeks 7, 15, 18, 23, 33, and 51 of the year are labeled as A through F respectively. Color coding and legend reflect relative probability (0.00–1.00) of H5 or H7 occurrence in North American Poultry with darker colors signifying increased likelihood. Note that C representing week 18 of the year (late April – early May) was predicted to exhibit the greatest AIP probability.

Of particular interest in Tables 5 and 6 are the shared spatial fields (${Z}_{shared}$) that represent the relationship of waterfowl telemetry to AIP occurrence probability. The shared fields for both the waterfowl residence time and occurrence probability joint models are estimated to be positive, with 95% credible intervals that exclude zero. In the instance of Model6, the ${Z}_{shared}$ estimate is interpreted to indicate that if the probability of waterfowl occurrence at a location is 0.5, then the probability of an AIP is greater than 0.5 while controlling for all other model covariates. More precisely, at locations having a 0.5 waterfowl occurrence probability, the chance of an AIP is 53.2% [(1/(1 + exp(0.13))) × 100%] while controlling for all other covariates. As the occurrence probability of waterfowl increases above 0.5, the chance of an AIP likewise increases above 53.2%. In the case of Model5, the ${Z}_{shared}$ estimate translates to a 63.6% [(1/(1 + exp(0.56))) × 100%] chance of an AIP at locations exhibiting the domain-wide mean waterfowl residence time of 8.6 days while holding all else constant. A greater AIP likelihood is expected for locations with longer waterfowl residence time. Combining all covariates, AIP probability can be estimated for any week of the year and any location within our study area; Fig. 7 displays the relative chance of an AIP for North America for select weeks of the year.

Model validation results are summarized in Table 7, which provides the PCC, Sensitivity, Specificity, AUC, and TSS for the hold-out data randomly partitioned during preliminary analysis. Statistics are provided for the three AIP presence thresholds determined to maximize Sensitivity, Specificity, and the TSS. To supplement Table 7, Table 8 further describes the validation dataset by providing the count of each virus subtype retained for evaluation as well as the average estimate and probability of cluster membership at each location.

**Table 7:** Model validation summary.

Threshold	PCC	Sensitivity	Specificity	AUC	TSS
0.50	0.81	0.95	0.67	0.96	0.61
0.74	0.90	0.89	0.91	0.96	0.80
0.76	0.90	0.88	0.91	0.96	0.79

Percent Correctly Classified (PCC) at given Threshold. Model sensitivity, specificity, and the AUC are scaled from 0 to 1. The TSS score is ranged from −1 to 1. Higher scores signify improved performance for all metrics.

**Table 8:** Predictive accuracy by virus subtype.

Subtype	Estimate (95% CI)	Cluster (95% CI)	Count
H5N1 HPAI	1.00 (1.00, 1.00)	0.99 (0.99, 0.99)	1
H5N1 LPAI	0.98 (0.98, 0.98)	0.79 (0.79, 0.79)	1
H5N2 HPAI	0.90 (0.85, 0.94)	0.61 (0.52, 0.69)	46
H5N2 LPAI	0.72 (−0.34, 1.79)	0.67 (−0.10, 1.44)	3
H7N1 LPAI	0.97 (0.97, 0.97)	0.79 (0.79, 0.79)	1
H7N3 HPAI	0.87 (0.80, 0.95)	0.76 (0.66, 0.85)	38
H7N3 LPAI	0.78 (−1.98, 3.54)	0.80 (−1.67, 3.26)	2
H7N8 LPAI	0.80 (0.14, 1.46)	0.51 (−0.76, 1.77)	2
H7N9 LPAI	0.72 (0.29, 1.15)	0.52 (−0.08, 1.11)	3

Model estimated event probability (Estimate), probability that the event belonged to a cluster (Cluster), and the count of testing samples randomly selected from original dataset.

## Discussion

Considering forty-two marked blue-winged teal as representative natural host waterfowl, we applied advanced temporal modeling techniques to evaluate the location and timing of waterfowl movement and space-use (1) relative to avian influenza events (AIP) across North America between 2004 and 2019 and (2) confirmed as resulting from viruses having the H5 or H7 hemagglutinin surface protein. While our primary goal was to utilize multi-year animal movement telemetry and disease event data to characterize intra-annual patterns, we also emphasized methodological facets to guard against attributing avian influenza detections to waterfowl when they are better explained by mere spatial proximity or membership to the AIP clusters in our sample. To accomplish these goals, we developed a dynamic joint (waterfowl-AIP) temporal model with shared components. Our combined animal movement and disease distribution framework was sufficiently flexible to accommodate a large study domain and enabled us to account for cumulative error across what might typically be approached as individual or separate analyses.

Results indicate that the presence of migratory waterfowl is positively related to AIP occurrence within North America. As waterfowl occurrence probability or residence time increase at a given location, so too does the chance of an AIP. This conclusion is supported by estimates for the shared spatial fields (see, ${Z}_{shared}$, Tables 5 and 6), which were specifically implemented to account for spatial interaction between waterfowl and AIP locations. The 95% credible intervals for the shared fields included with both the waterfowl residence time (Model5) and occurrence probability (Model6) models indicate that waterfowl presence improves the model’s ability to estimate AIPs and that inclusion of movement telemetry increases parsimony (Table 4). Importantly, our inferred support for waterfowl improving AIP estimation is made after accounting for the week-specific, latitudinal displacement of migratory birds (Model3), poultry abundance (Model4), and AIP clustering (Model2). While our approach indicates that waterfowl presence and residence time were associated with North American AIPs during 2004–2019, we cannot conclude that any individual AIP was the direct result of wild bird to poultry viral spillover. Given that HPAI phenotypes are recognized as only evolving in domestic poultry, it is probable that associations between waterfowl presence and AIPs are a function of LPAI H5 and H7 spillover from wild birds to domestic poultry, or that the spatiotemporal associations resulted from other unidentified and unmeasured factors.

Regarding AIP clusters, we speculate that not all^69^ of the clustered AIPs visibly apparent (Fig. 2) over portions of Mexico (e.g., Jalisco) and the upper Midwestern U.S. (e.g., Minnesota) represent independent introductions of avian influenza by wild birds. Given the compact spatial arrangement of the events, relative incident timing (Fig. 3)^70,71^, and the commonality of virus subtypes at each location (e.g., H7N3 in Jalisco, H5N2 in Upper Midwest), we conjecture that AIP clusters likely stemmed from some combination of mechanical (i.e., shared farm equipment, staff movement between farms, etc.) and environmental (i.e., common water, soil, and air) transmission^28,72^. This interpretation is consistent with recent genetic analyses showing viral relatedness among H5N2 HPAI outbreaks in the Midwest^26,69^ and among H7N3 HPAI outbreaks in Mexico^17^. In the absence of extensive genetic sampling and lacking information detailing farm-specific operational practices, the current study is insufficient in itself to assess the tenability of a “farm-to-farm” or “lateral spread” disease cluster hypothesis directly, but, from a modeling context we propose Tobler’s Law^73^ as an accessible heuristic to account for the spatial patterns that sometimes arise from disease and epidemiological processes. The constructed covariate we designed to quantify fine-scale spatial structure is a statistical implementation of Tobler’s Law in that it assigns a cluster membership probability to all locations in the study area based on proximity to a neighboring AIP. To paraphrase Tobler from an epidemiological perspective, we assumed that “nearer outbreaks are more related than distant outbreaks”. If a detection is better explained by being in close proximity to another AIP than it is by waterfowl occurrence, then the risk of inferring an independent introduction by waterfowl is inherently reduced.

The timing of seasonal waterfowl migration was also found to be an AIP indicator. The time-structured covariate reflecting bird net latitudinal displacement was important as shown by 95% credible intervals (Fig. 6) and improved model parsimony (Model3, Table 4). Model results suggest that AIP occurrence probability is greatest during the months of April and May, a period coincident with peak blue-winged teal northward movement, and less during the months of September and October, which is the peak of observed southward movement. Stated differently, the chance of an AIP occurring in North America is greatest during the spring migration, less during the fall migration, and generally insignificant during the waterfowl breeding and overwintering seasons. More generally, the temporal relationship of waterfowl to past AIPs suggests that the chance of avian influenza being introduced into a U.S. poultry facility steadily increases between February and May and then rapidly decreases for the remainder of the year. Although increased hatchling abundances and bird aggregation at pre-migration staging areas through the late-breeding and fall seasons (August and September) are thought to contribute to a generally higher AI prevalence in dabbling ducks broadly, this spatiotemporal pattern may not be as consistent for blue-winged teal specifically. Having a reputation for early departure and long flight distances, vast numbers of blue-winged teal have already vacated pre-migration staging areas and arrived at migration stop-over sites or overwintering habitats by August and September^40,74^. Having experienced reduced rates of virus exposure prior to autumn migration, immunologically naïve blue-winged teal may help maintain AI at lower latitude locations throughout the non-breeding period and potentially redistribute virus during the spring migration^40,41,75^. As it relates to both the timing and degree of clustering shown in our AIP sample, it is important to note that models underestimate AIP probability over several areas in the upper Midwest and northwestern U.S., as well as in central Mexico (Fig. 4(C,D)). This indicates that additional factors or variables are needed to fully explain the epidemiological processes at these locations. Remaining error may be due to delayed surveillance or event reporting, environmental persistence, post-introduction redistribution by wild birds, farm-specific operational practices, species-specific virus relationships, or other unmodeled factors.

Geographically, several regions appear particularly vulnerable to an AIP. In the U.S., the states of Mississippi, Alabama, and Georgia in the Southeast exhibit elevated risk from February through April (Fig. 7). The Delmarva Peninsula on the East Coast shows an increased chance of a poultry influenza event during the second half of March through April as does the southern Great Lakes Region and central California on the West Coast. By comparison to the U.S., model estimates indicate relatively lower AIP probabilities for southern Canada and Mexico. Although Southwestern Ontario and the Montreal vicinity show increased risk at the height of waterfowl spring migration (Fig. 7(C)), AIP suitability in Canada falls below 15% for the remainder of the year. In Mexico, the highest AIP probability was estimated for the second-half of January and first part of February when conditions were approximately 30% suitable for an AIP along the western-most edge of the Mexican Plateau between Jalisco in the north and Puebla to the south. This estimated period of highest AIP probability in Mexico coincides with the timing of blue-winged teal staging for the spring migration.

Although validation evidenced overall good model performance (Tables 7 and 8), comparing model estimated AIP probabilities to the full disease occurrence dataset used as input (Fig. 2) suggests that the model’s ability to capture past AIPs varied by virus subtype. More specifically, waterfowl space use and migration timing show greater correspondence to past H7 AIPs than to those of H5 origin. The majority of H5 events (e.g., H5N2 and H5N8) incorporated into our analyses were sampled from the outbreaks in 2014–2015 that affected 21 States across the Northwestern and Midwestern U.S.^27^, but, as discussed with respect to possible farm-to-farm transmission above, estimated AIP occurrence across these regions is generally very low (Fig. 7) and considerable residual error remains (Fig. 4) at these locations. By comparison, AIP probabilities in the vicinity of past H7 events (e.g., H7N3 and H7N9) are considerably higher. This tendency may be a reflection of blue-winged teal being more commonly infected with H7 subtype viruses during spring as compared to H5 viruses^18^. In short, model results provide additional support that blue-winged teal were unlikely acting as a vector of HPAI during the 2014–2015 H5N2 and H5N8 outbreaks across the U.S.^71^; however, findings may add support to the notion that this species could introduce H7 subtype viruses to domestic poultry during the spring migration.

Our study could be improved in at least a couple regards. First, despite having intended our marked blue-winged teal as representative of the larger North American dabbling duck population, we acknowledge that migratory movement behavior may vary even within a species and that our study likely exhibits species-specific sample bias. As discussed above, the tendency for blue-winged teal to be early and long-distance flyers may have important implications for AI disease ecology and the representativeness of the species as an archetypal dabbling duck. We likewise recognize that bird-virus associations are often species-specific due to co-adaptation and variable physiological and immunological tolerances. Studies drawing on a larger sample size or incorporating different duck species under a comparative framework may be better suited to infer waterfowl-AIP relationships over such a heterogeneous study area. Studies aimed at investigating waterfowl-AIP dynamics by either choosing a wider range of focal bird species or being more selective in the virus subtypes examined would certainly be worthwhile. Secondly, the analysis of a large spatial domain may offer insight into long-distance animal movement and virus transmission potential, but, it also restricts data precision and availability. For example, the Gridded Livestock database that was used to estimate poultry abundance was essential to our study, but, had focused on a smaller geographic area in the U.S., we would have gained access to more recent and detailed information to characterize poultry locations and abundances.

In closing, we believe that the spatial and temporal relationship of blue-winged teal to past AIPs offers important insights into migratory waterfowl ecology and avian influenza disease dynamics that can aid in better preparing for future AIPs. Being able to anticipate the geographic locations and times most vulnerable to avian influenza outbreaks would be an asset to the domestic poultry industry and those oversight agencies responsible for disease surveillance and response. Additionally, we hope that the methodological approach presented in this study will motivate future research into modeling disparate facets of disease systems at the wildlife-agriculture interface.

## Data Availability

All analysis and modeling were performed using the open-source R language for statistical computing^52^ with freely-available data. Wild bird telemetry, environmental data, and avian influenza detections can be accessed using web addresses provided in the Materials and Methods Section.
